# Anisotropic Mechanical Properties of 3D Printed Low-Carbon Concrete and Connection Strategies for Large-Scale Reusable Formwork in Digital Construction

**DOI:** 10.3390/ma19010145

**Published:** 2025-12-31

**Authors:** Binrong Zhu, Miao Qi, Wei Chen, Jinlong Pan

**Affiliations:** 1 Jiangsu Provincial College Key Laboratory of Intelligent Bridge Construction and Safe Operation & Maintenance, College of Civil Engineering, Nanjing Forestry University, Nanjing 210037, China; 2 School of Civil Engineering, Southeast University, Nanjing 211189, China; 18834200019@163.com; 3 School of Civil and Architectural Engineering, Guizhou University of Engineering Science, Bijie 551700, China; 15293160426@163.com

**Keywords:** 3D concrete printing, low carbon concrete, reusable formwork, connection strategy

## Abstract

3D concrete printing (3DCP) is an emerging intelligent construction technology that enables the direct transformation of digital models into physical components, thereby facilitating the precise fabrication of complex geometries. This study investigates the anisotropic mechanical properties and construction applicability of low-carbon 3D printed concrete for reusable formwork systems. Axial compression, flexural, and splitting tensile tests were conducted to examine mechanical anisotropy, and the effects of steel slag and iron tailings replacement levels on mechanical performance were evaluated. Carbon emission analysis was also performed. Using the coefficient-of-variation TOPSIS method, an optimal printable low-carbon mixture was identified, comprising 30% steel slag, 40% iron tailings sand, and 0.3% fibre content, balancing both mechanical performance and environmental benefits. To address the challenges associated with printing large monolithic formwork units, such as excessive weight and demoulding difficulties, three connection strategies for curved wall modular reusable formwork were designed. Finite element analyses were conducted to assess the strength and stiffness of each strategy, and an optimized connection configuration was proposed. The findings demonstrate the feasibility of accurately fabricating complex architectural components using low-carbon 3D printed concrete, providing theoretical and practical support for the industrialized production of large-scale, geometrically complex structures.

## 1. Introduction

3D printing technology, also referred to as additive manufacturing, originated in the late 1980s as an innovative rapid fabrication technique [1]. Its fundamental principle lies in transforming a digital model into a physical object through a layer-by-layer deposition process, enabling the accurate fabrication of geometrically complex structures [2]. As urbanization and industrialization accelerate worldwide, the construction sector is increasingly challenged by labour shortages, resource constraints, low productivity, and safety concerns. Against this backdrop, the emergence of 3D concrete printing (3DCP) provides a promising technological pathway for addressing these long-standing issues [3,4,5]. Compared with conventional construction methods, 3DCP offers several significant advantages, including formwork-free fabrication, enhanced geometric freedom, high production efficiency, and improved potential for functional integration and mass customization.

At the current stage of development, 3D printable concrete mixtures primarily consist of cementitious binders and fine aggregates, typically without the inclusion of coarse aggregates. This composition leads to a significantly higher cement content than that used in conventional cast concrete [6]. However, cement production is energy-intensive and associated with substantial CO_2_ emissions, posing a major obstacle to meeting the objectives of green and low-carbon construction [7]. Consequently, partially replacing cement with industrial by-products or solid wastes has become a critical strategy for reducing binder demand and improving the sustainability of 3D printing materials. In addition, the fine aggregates used in 3D printable concretes are predominantly derived from natural river sand. Continuous high-intensity extraction over recent decades has resulted in severe depletion of natural sand resources [8,9,10,11]. The rapidly increasing demand for sand in the construction industry has further intensified supply shortages, while excessive riverbed mining has caused ecological problems such as channel degradation and riverbank instability. Therefore, the incorporation of suitable natural-sand alternatives into 3DCP has become an urgent requirement for advancing its engineering implementation and sustainable application.

Among the various sustainable materials considered for developing low-carbon 3D printable concrete, steel slag and iron tailings have attracted increasing attention due to their large reserves and compatibility with cementitious systems. Steel slag, characterized by latent hydraulic or pozzolanic activity, can serve as a supplementary cementitious material to partially replace Portland cement, thereby reducing binder-related carbon emissions while contributing to strength development [12,13]. In contrast, iron tailings possess a stable mineral composition, low impurity content, and a fine particle gradation, which make them suitable substitutes for natural river sand [14,15,16,17]. Incorporating these industrial by-products into 3D printed concrete not only diverts large volumes of solid waste from landfills but also helps reduce the demand for natural sand and the environmental burden associated with cement production, thereby supporting the development of low-carbon 3DCP materials. Although previous studies have demonstrated the feasibility of incorporating industrial by-products into cementitious systems, most existing research on 3D printed concrete has primarily focused on conventional binders and aggregates, with limited attention given to steel slag and iron tailings under extrusion-based printing conditions, particularly in terms of anisotropic mechanical performance. However, research specifically focusing on their application in 3D printable low-carbon concrete remains limited. In particular, the hardened-stage anisotropic mechanical behaviour arising from the inherent layer-by-layer deposition characteristic of 3DCP has not yet been systematically investigated. Therefore, exploring the fundamental hardened-stage anisotropic mechanical properties of 3D printed low-carbon concrete incorporating steel slag and iron tailings is essential for ensuring reliable structural performance and enabling its broader engineering implementation.

In recent years, the pursuit of greater geometric freedom and construction efficiency has motivated substantial global research efforts to overcome the inherent limitations of planar formwork in conventional concrete construction. Emerging studies have explored two main pathways for fabricating non-standard, doubly curved, or topology-optimized moulds: (1) reusable formwork systems derived from flexible or granular materials through hybrid additive–subtractive manufacturing; and (2) permanent formwork fabricated directly using 3D-printed cementitious composites. Reusable formwork research has demonstrated promising potential for reducing material consumption and enabling rapid forming of complex geometries. Examples include pneumatic formwork designed through two-dimensional sealing-pattern optimization [18], recyclable sand–binder composite formwork produced via subtractive milling [19], and highly flexible thermoplastic polyurethane (TPU) moulds additively manufactured for precast façade elements [20]. These techniques collectively highlight the adaptability, environmental benefits, and reusability of alternative formwork strategies, while also revealing current limitations such as size dependence on material width, high production costs, and sensitivity to assembly or casting procedures.

Parallel to reusable systems, 3D-printed permanent formwork has gained increasing attention due to its exceptional geometric adaptability and ability to integrate with cast-in-place or precast concrete. Early studies have shown that fibre-reinforced or fabric-reinforced cement-based permanent formwork can significantly enhance interfacial bonding, composite action, and structural deformation capacity [21,22]. Subsequent applications, such as customized 3D-printed stair formwork, have further demonstrated advantages in fabrication precision and material efficiency [23]. Nevertheless, several challenges remain. These include the limited feasibility of arranging reinforcement in complex geometries, the difficulty of ensuring high-quality casting within intricately printed cavities, and the fact that permanent formwork cannot be reused, which ultimately increases life-cycle costs. Consequently, integrating the geometric freedom of 3D-printed cementitious materials with the reusability concept offers a compelling pathway toward low-carbon, large-scale, recyclable construction templates. Such a hybrid strategy could substantially improve environmental performance, reduce material consumption, and support the digital construction of complex building components, thereby motivating deeper investigation into the anisotropic mechanical behaviour and connection design of 3D-printed low-carbon concrete templates.

Based on the integration of 3D printing technology with building industrialization, this study investigates the fundamental mechanical behaviour of 3D printed low-carbon concrete and its application in reusable concrete formwork for complex architectural components. Anisotropic compressive, flexural, and splitting tensile tests were conducted on mixtures incorporating different proportions of steel slag and iron tailings, with cast concrete used as a reference. The effects of replacement ratios on mechanical performance, anisotropy, and carbon emissions were quantified, and the coefficient-of-variation TOPSIS method was employed to identify the optimal low-carbon printable mixture. Furthermore, to address the excessive weight, transportation difficulty, and demoulding challenges associated with printing large monolithic formwork units, modular connection strategies were examined. Lateral pressure exerted by fresh concrete was determined according to relevant standards, and ABAQUS finite element simulations were performed to evaluate stress and deformation under various connection configurations. Comparison with design limits enabled the selection of a feasible connection strategy for reusable 3D printed low-carbon concrete formwork. It should be noted that this study primarily concentrates on mechanical anisotropy, carbon performance, and structural applicability of 3D printed low-carbon concrete, while microstructural mechanisms and long-term durability aspects are not addressed in detail. Further investigations are therefore required to systematically explore the durability behaviour and micro–macro interactions of such materials in 3D printed concrete systems.

## 2. Materials and Methods

### 2.1. Raw Materials and Mix Proportion

**Figure 1:**
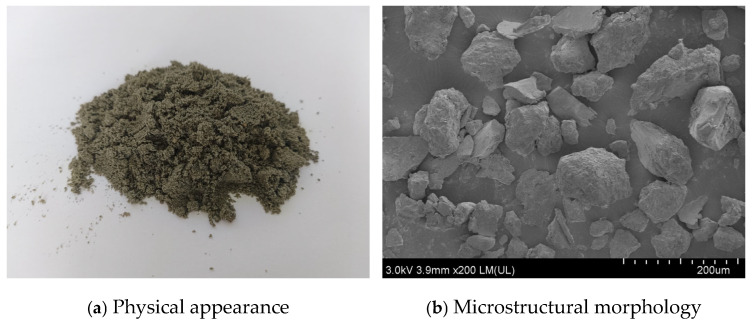
Morphology of the iron tailings sand.

In this study, the cementitious system of the 3D printed low-carbon concrete consisted of P·O 42.5 ordinary Portland cement, grade 955 silica fume (density 1.57 g/cm^3^), steel slag (specific surface area 2.214 m^2^/g), and class I fly ash (density 2.34 g/cm^3^). The fine aggregates were river sand and iron tailings sand, both with particle sizes below 1.18 mm. The iron tailings sand was mainly composed of SiO_2_ and featured porous, irregularly shaped particles with a rough surface and good dispersion, as shown in Figure 1.

Short polypropylene (PP) fibres with a length of 6 mm were used to reinforce the matrix, and their main properties are listed in Table 1. Owing to their high tensile strength, elasticity, and durability, PP fibres effectively reduce cracking caused by autogenous shrinkage [24,25]. A polycarboxylate-based superplasticizer with a water-reducing efficiency above 30% was added to adjust the flowability of the mixture to meet extrusion requirements. Tap water at room temperature was used for mixing.

**Figure 2:**
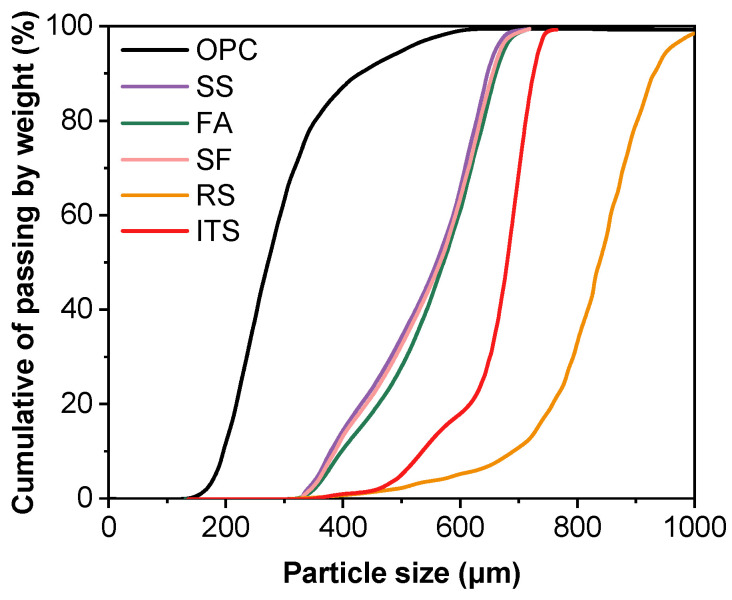
Particle size distribution of OPC, SS, FA, SF, RS and ITS.

The particle size distributions of cement, silica fume, steel slag, fly ash, iron tailings sand, and river sand were measured using a laser diffraction analyser (Mastersizer 2000, Malvern Panalytical Ltd., Malvern, UK) (Figure 2). The mix proportions of different groups are summarized in Table 2 and denoted as 0.1-0.2-0%, 0.2-0.6-0.3%, 0.3-0-0.9%, 0.3-0.2-0.6%, and 0.3-0.4-0.3%, where the first, second, and third numbers represent the replacement ratios of steel slag, iron tailings sand, and fibre content, respectively. It should be noted that the mix proportions listed in Table 2 were preliminarily selected based on prior orthogonal testing and were further confirmed to meet the printability requirements. The corresponding printability results are presented in Section 2.3.

### 2.2. Specimen Description

The preparation process of the 3D printed low-carbon concrete specimens was as follows. All dry materials (OPC, steel slag, fly ash, silica fume, river sand, and iron tailings sand) were first placed in a 60 L horizontal mixer and blended at 60 rpm for 2 min. Afterward, the predetermined amount of water was added and mixed slowly at 60 rpm for 3 min. Polypropylene (PP) fibres were then gradually introduced into the mixture and blended at 100 rpm for 1 min to ensure uniform dispersion. The total mixing time was maintained at 6 min. The freshly mixed material was subsequently loaded into the hopper of the 3D printer, and the specimens were fabricated through continuous extrusion following the preset printing programme.

**Figure 3:**
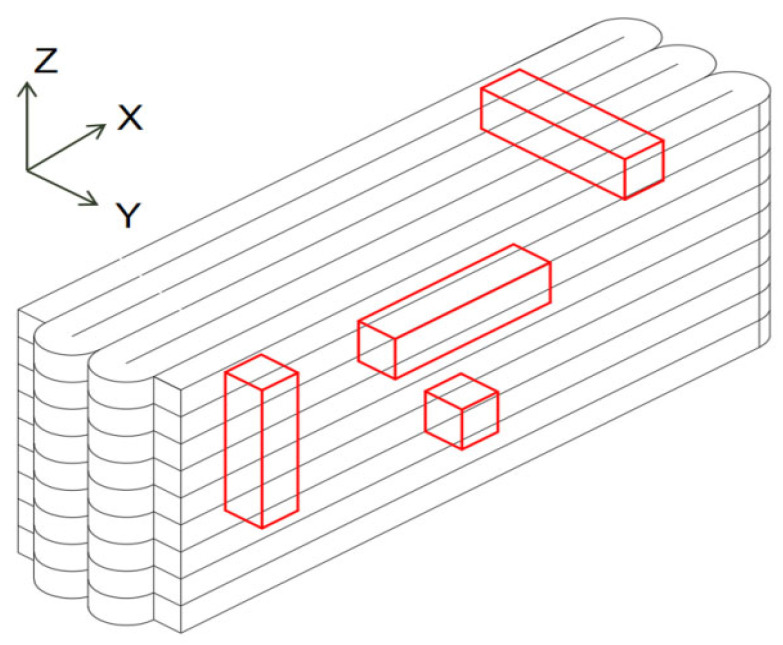
Printing and cutting diagrams for mechanical test specimens.

**Figure 4:**
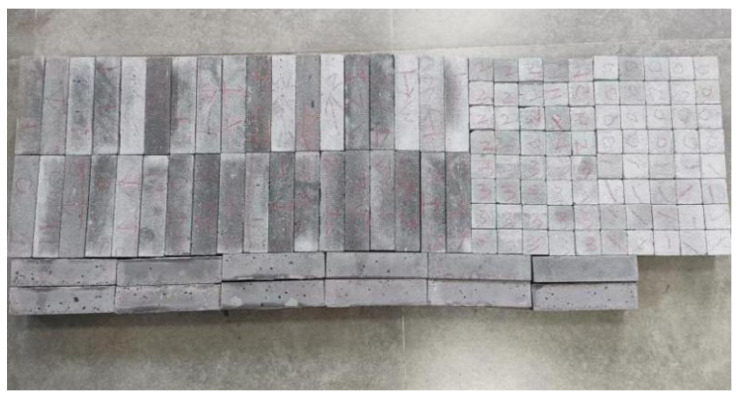
Test specimens for mechanical properties of 3D-printed concrete after cutting.

According to the Chinese standard GB/T 17671-2021 [26], 40 × 40 × 40 mm cubes were prepared for compressive and splitting tensile strength tests, and 40 × 40 × 160 mm prisms were prepared for flexural strength tests. After printing, the specimens were left in ambient laboratory conditions for 1 day and then cut into the required dimensions using a concrete cutting machine. The cut specimens were immediately transferred to a standard curing chamber (20 ± 2 °C, relative humidity 95% ± 5%) until the designated testing age. Figure 3 and Figure 4 show the specimen cutting procedure and the prepared test specimens.

### 2.3. Three-Dimensional Concrete Printing

**Figure 5:**
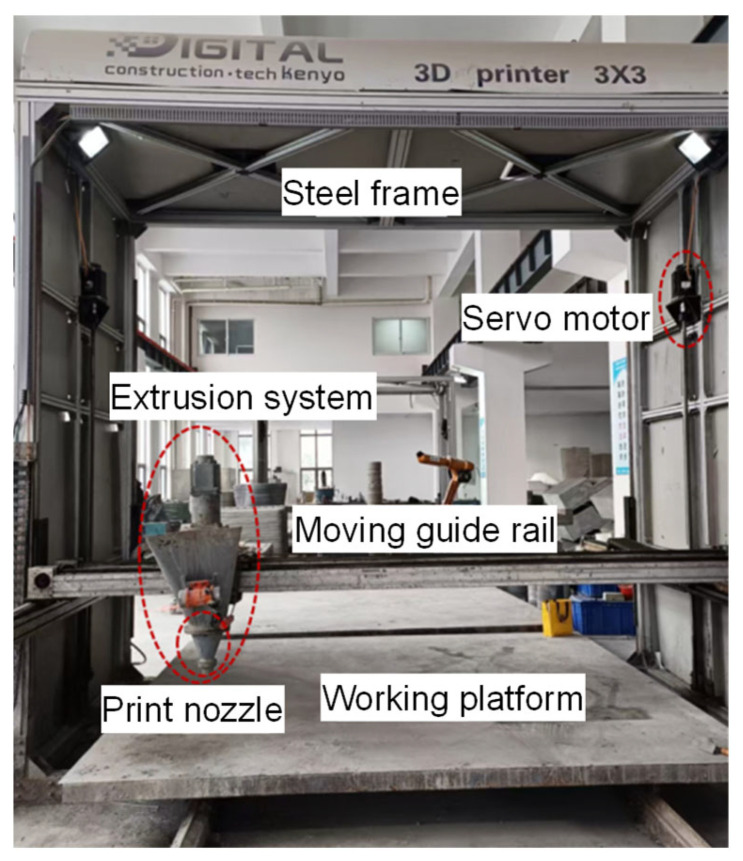
Gantry-type 3D concrete printer.

A screw-extrusion-based 3D concrete printer with a geometric printing range of 3 × 3 × 2 m was used in this study (Figure 5). The printer consists of three main modules: the frame system, the working platform, and the printing system. The printing system is composed of three subsystems: the control, motion, and extrusion systems. The control system integrates computer-based motion control software and extrusion parameter adjustment modules, enabling real-time control of the nozzle’s spatial positioning and extrusion rate. The motion system adopts a framed structure with *x*–*y*–*z* linear guides and lead screw drives. The extrusion system comprises a feed hopper, a metering pump, and a printing nozzle. Driven by a servo motor, the screw-type metering pump continuously delivers the mixed material to the nozzle, where it is uniformly extruded and deposited layer by layer along the programmed toolpath to form the designed 3D structure. The nozzle travel speed and screw rotation speed were set to 9 m/min and 60 r/min, respectively.

Each mixture was evaluated for printability, which requires both extrudability and buildability. The extrudability criteria included: (1) the material must be extruded continuously without interruption or segregation; (2) the extruded filaments should have smooth and compact surfaces without visible pores or cracks; and (3) the deviation between the measured and designed filament widths should not exceed ±5 mm [27]. Continuous filaments of 2000 mm in length were printed to observe surface integrity and dimensional stability. Buildability was assessed by printing stacked elements with a designed length of 600 mm and a height of 180 mm. The material was required to maintain its shape and structural stability during layer deposition, ensuring no collapse, tilting, or delamination between layers. The printed structures were expected to sustain their self-weight and minor external disturbances during printing [28]. The layer deformation and vertical displacement were measured to evaluate buildability.

The results of the extrudability and buildability tests are summarized in Table 3. All mixtures exhibited stable and continuous extrusion without cracking or segregation, and the printed filaments had smooth, dense surfaces with width deviations within ±5 mm. No visible deformation or instability occurred during the deposition process, and all mixtures were capable of stable deposition up to 15 layers, meeting the technical requirements for 3D printable concrete. In addition, rheological tests were conducted using a Brookfield rheometer (RST-SST, AMETEK Brookfield, Middleboro, MA, USA) on all 5 mixtures. The results indicate that the dynamic yield stresses of the mixtures 0.1-0.2-0%, 0.2-0.6-0.3%, 0.3-0-0.9%, 0.3-0.2-0.6%, and 0.3-0.4-0.3% were 182.72 Pa, 397.06 Pa, 146.57 Pa, 162.93 Pa, and 220.32 Pa, respectively. All values fall within the commonly reported suitable range for extrusion-based 3D printable concrete, which is approximately 100 to 800 Pa [29].

In summary, the selected concrete mixtures exhibited stable extrusion performance and excellent shape retention during the printing process. These results indicate superior extrudability and buildability, fully meeting the technical requirements for 3D printing construction. Therefore, the selected mix design can serve as a reference formulation for subsequent studies on material performance optimization and engineering applications.

### 2.4. Experimental Set-Up and Measurements

The 3D printed concrete specimens were categorized into three loading directions according to the printing orientation, as illustrated in Figure 3. Compressive, flexural, and splitting tensile strengths measured along these directions were denoted as Fx, Fy, and Fz, corresponding to the directions parallel to the printing path, perpendicular to the printing path, and vertical to the deposition layers, respectively. All tests were conducted in accordance with the Chinese standards T/CBMF 183-2022 [30] and GB/T 17671–2021 [26].

#### 2.4.1. Mechanical Property Testing

**Figure 6:**
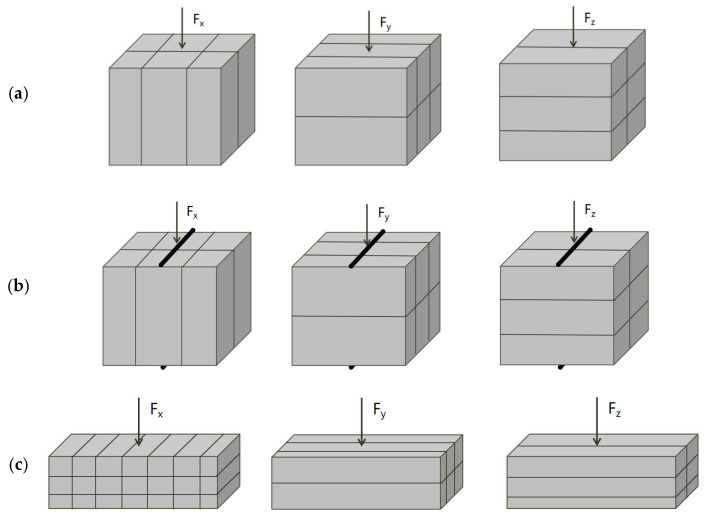
Mechanical property test loading configurations: (**a**) compressive test; (**b**) splitting tensile test; (**c**) flexural test.

Mechanical testing, including compressive, splitting tensile, and flexural strength tests, was performed using an electronic universal testing machine, as shown in Figure 6. For each loading direction, three standard specimens were prepared and tested after 28 days of curing. The mean value of the three specimens was taken as the representative strength for that direction. The strength of the 3D printed samples was also compared with that of mould-cast specimens having the same mix proportions.

During the compressive test, a loading rate of 1.5 kN/s was applied. For the splitting tensile test, loads were applied to the upper and lower surfaces of the specimen through steel strips at a rate of 50 N/s. The three-point bending test was carried out under a loading rate of 10 N/s. The compressive strength (*f_c_*), splitting tensile strength (*f_tb_*), and flexural strength (*f_f_*) were calculated using Equations (1)–(3):

(1) $$f_{c}=\frac{P_{f}}{A}$$

(2) $$f_{tb}=\frac{2P_{u}}{\pi A_{t}}=0.637\frac{P_{u}}{A_{t}}$$

(3) $$f_{f}=\frac{3F_{f}L}{2bh^{2}}$$

where *P_f_* is the peak load at failure (N), and *A* is the loaded area (mm^2^); *P_u_* is the ultimate load (N), and *A_t_* is the loaded cross-sectional area (mm^2^); *f_f_* is the flexural strength (MPa), *L* is the span length (m), and *b* and *h* are the specimen width and height (mm), respectively.

#### 2.4.2. Anisotropy Evaluation

To quantitatively assess the mechanical anisotropy of 3D printed concrete, Ye et al. [31] refined the empirical formula originally proposed by Ma et al. [32], emphasizing that anisotropy should be evaluated by comparing specimens manufactured under identical printing conditions. Based on this concept, the directional anisotropy index *I_Direction_* is calculated using Equation (4). In this study, the overall mechanical anisotropy of the 3D printed low-carbon concrete is expressed as the average anisotropy index of the three principal loading directions [33], as shown in Equation (5).

(4) $$I_{Direction}=\sqrt{{\left(f_{X}-f_{Direction}\right)}^{2}+{\left(f_{Y}-f_{Direction}\right)}^{2}+{\left(f_{Z}-f_{Direction}\right)}^{2}}/f_{Direction}$$

(5) $$I_{Mechanical}=\left(I_{X}+I_{Y}+I_{Z}\right)/3$$

where *f_X_*, *f_Y_*, and *f_Z_* are the mechanical strengths of the 3D printed concrete under loading in the *X*-, *Y*-, and *Z*-directions, respectively. *I_Direction_* represents the anisotropy index corresponding to a specific loading direction, and *f_Direction_* denotes the strength in that direction. *I_Mechanical_* is the average anisotropy value representing the overall mechanical anisotropy of the material.

#### 2.4.3. Carbon Emission Calculation

At present, three mainstream approaches are used for carbon accounting: the emission factor method, the mass balance method, and the direct measurement method. Among them, the emission factor method is the most widely applied. It estimates carbon emissions based on the amount of energy or material consumed and the corresponding emission factors, following the basic form: Carbon emissions = Consumption × Emission factor. The full life cycle of ready-mixed concrete production involves five key stages: raw material extraction and processing, raw material transportation, concrete mixing, transportation of finished concrete, and on-site construction [34].

The direct and indirect emissions generated through these stages can be categorized into three main components: (1) Emissions from raw material production and transportation (*C*_1_), including emissions from material production (*C*_11_), raw material transport (*C*_12_), and concrete mixing (*C*_13_). (2) Emissions from delivering mixed concrete to construction sites (*C*_2_). (3) Emissions generated during on-site construction activities (*C*_3_). The present study adopts a cradle-to-gate boundary focused on the concrete production stage, which includes raw material production, raw material transportation, and concrete mixing. Emissions associated with on-site construction, service life, demolition, and recycling are outside the scope of this study. The carbon emissions generated from raw material production are calculated using:

(6) $$C_{11}=\sum C_{i}m_{i}$$

where *C_i_* is the emission factor of each raw material, and *m_i_* is the corresponding material dosage.

In addition to production-related emissions, raw material transportation also contributes significantly to the total carbon footprint, with transport distance being the primary influencing factor. Due to differences in production sites, transport distances vary among materials. In practical projects, cement and natural aggregates are typically sourced from nearby suppliers to minimize transportation emissions. The carbon emissions associated with transportation are calculated as:

(7) $$C_{12}=\sum m_{i}S_{i}F$$

where *S_i_* is the transport distance for each raw material, and *F* is the emission factor for road transportation.

The carbon emissions during the concrete mixing stage mainly arise from energy consumption and equipment operation. In this study, only the electricity consumption of the mixer is considered, and the emissions associated with concrete mixing are calculated as:

(8) $$C_{13}=O\times D$$

where *O* is the electricity consumption per unit mass of concrete and *D* is the emission factor for electricity.

#### 2.4.4. Connection Strategies for Printed Formwork

To ensure the decorative performance of the 3D printed curved wall reusable formwork while reducing its self-weight, the formwork surface was modularized according to the geometric pattern. This strategy reduces the required support system, improves production efficiency, and facilitates transportation and demoulding. Three connection strategies were designed and evaluated in this study.

**Figure 7:**
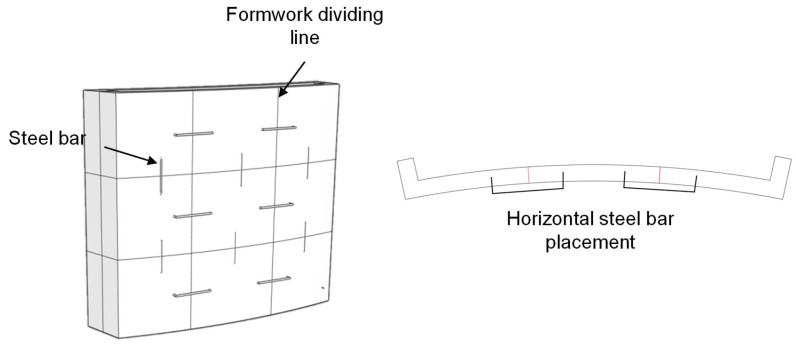
Schematic of formwork connection Strategy 1.

Strategy 1: The formwork was divided into longitudinal and transverse segments following the surface pattern. U-shaped steel bars were bent in advance and inserted into the reserved openings during assembly. Friction between the steel bars and the printed formwork, combined with the slight deformation of the bars under the lateral pressure of fresh concrete, produced a ring-hoop-like confinement effect that enabled effective connection. The connection configuration is illustrated in Figure 7.

**Figure 8:**
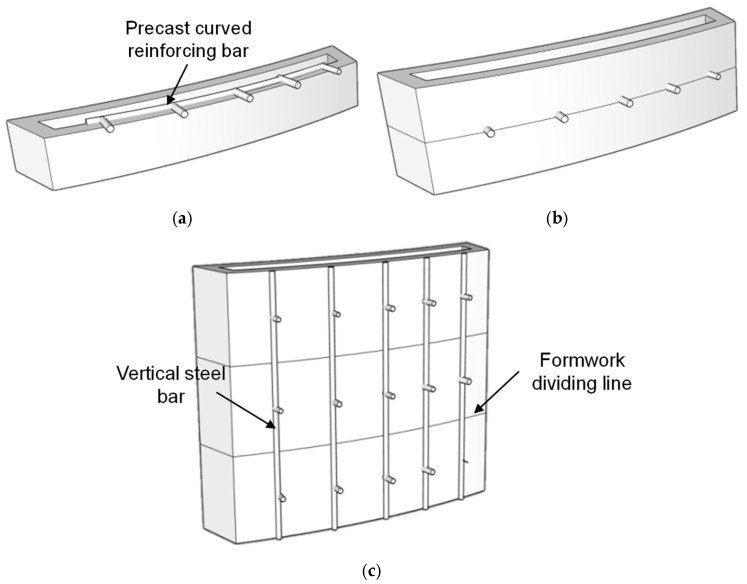
Schematic of formwork connection Strategy 2. (**a**) Insertion of prefabricated rebars during printing. (**b**) Embedding of rebars by printing the upper concrete layer. (**c**) Lap connection between vertical and embedded rebars.

Strategy 2: The formwork was divided longitudinally, and during the printing process, curved prefabricated steel bars matching the formwork profile were embedded at predetermined heights. These embedded bars were equipped with outward-extending welded ends at regular printing intervals. During assembly, vertical external reinforcement was connected to the protruding ends using lap joints, thus forming an integrated reinforcement system. The connection concept is shown in Figure 8.

This strategy creates an external longitudinal reinforcement system that works together with the internal embedded steel bars to resist lateral pressure during concrete casting and prevent horizontal displacement or deformation. However, it has practical limitations: it is mainly suitable for components with vertical external surfaces. For outwardly protruding geometries, the vertical steel bars must be bent precisely to match the curvature, which increases fabrication difficulty and accuracy requirements. This also leads to higher material consumption, construction cost, and significantly extended fabrication time.

**Figure 9:**
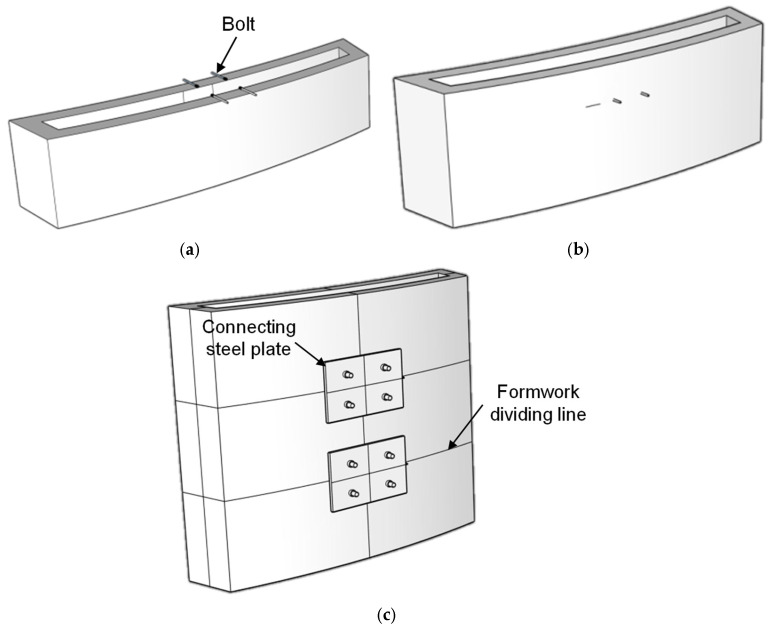
Schematic of formwork connection Strategy 3. (**a**) Insertion of bolts during printing. (**b**) Embedding of bolts via printing of the upper concrete layer. (**c**) Connection using perforated steel plates.

Strategy 3: The formwork was segmented in both vertical and horizontal directions. The lower layer of concrete was printed first, and bolts were embedded once the printing reached the designated height, followed by printing the upper layer. During assembly, perforated steel plates were used to complete the connections. Before the formwork hardened, the joint surfaces were levelled using a scraper to ensure proper seating for the steel plates. The connection configuration is illustrated in Figure 9.

## 3. Results and Discussion

### 3.1. Mechanical Properties

#### 3.1.1. Compressive Strength

**Figure 10:**
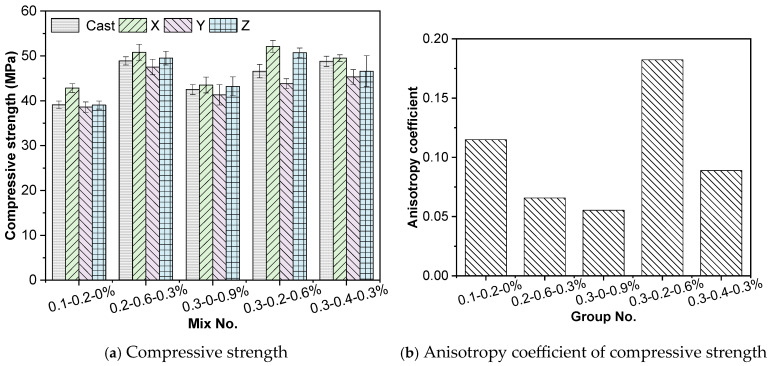
Compressive strength test results.

The compressive strength results of each mixture are presented in Figure 10. The 3D printed concrete exhibited pronounced anisotropy under different loading directions, consistent with observations reported in previous studies [35,36]. Among the three directions, the X-direction (parallel to the printing path) showed the highest compressive strength, followed by the Z-direction (layer deposition direction), while the Y-direction (perpendicular to the printing path) exhibited the lowest values. Compared with cast specimens of identical mix proportions, the compressive strength in the X-direction was higher by 9.5%, 3.9%, 2.4%, 11.8%, and 1.4%, whereas the Y-direction strength after 28 days was lower by 1.5%, 2.8%, 2.8%, 6.0%, and 7.1%, respectively.

Since the PP fibres used in this study are short and flexible, their influence on compressive strength is minimal. The results show that the compressive strength increases with the replacement ratios of steel slag and iron tailings sand. This trend is primarily attributed to the active constituents in steel slag, such as C_3_A and C_2_A, which participate in hydration and generate additional hydration products (e.g., Ca(OH)_2_, ettringite), thereby enhancing the cementitious matrix and improving strength. Although iron tailings sand has no pozzolanic activity, its fine and angular particles contribute to better packing density. As the replacement level increases, the improved gradation enhances compactness, and the high hardness of iron tailings provides additional skeletal support, collectively contributing to higher compressive strength.

The anisotropy indices for compressive strength remained below 0.20 for all mixtures, indicating relatively small directional differences. Overall, the anisotropy index decreased with increasing steel slag content. This is because anisotropy is influenced by the distribution of C–S–H gels across the interlayer interfaces. The addition of steel slag inhibits early-age C–S–H nucleation, but its sustained hydration at later stages increases gel formation and reduces anisotropy [12,37]. Conversely, replacing natural sand with angular iron tailings tends to increase anisotropy, as their irregular particle morphology leads to different packing and orientation in different printed directions, thus accentuating directional variation.

#### 3.1.2. Splitting Tensile Strength

**Figure 11:**
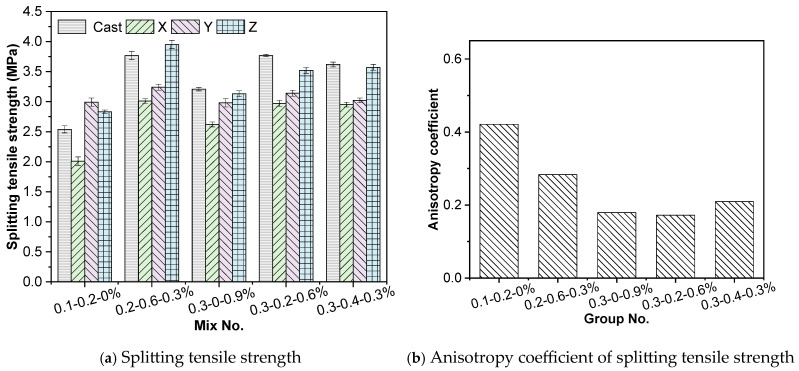
Splitting tensile strength test results.

The splitting tensile strength results of the specimens are shown in Figure 11. Overall, the 3D-printed low-carbon concrete exhibits clear anisotropy, following the trend Z-direction (layer deposition) > Y-direction (perpendicular to printing path) > X-direction (printing path). Under X-direction loading, the applied load is nearly parallel to the weak interfaces between layers, where stress concentration readily occurs, promoting premature crack initiation and propagation; thus, the lowest tensile strength is observed. In contrast, for Y- and Z-direction specimens, the load is applied perpendicular to the extruded filaments, delaying the development of dominant cracks and resulting in higher splitting strength.

A comparison across mixtures reveals that only the 0.1-0.2-0% mixture shows a notable reduction in splitting strength for the cast specimen relative to the 3D-printed Z-direction specimen, with a difference of approximately 10.25%. For all other mixtures, the splitting strength of the cast specimens is close to that of the 3D-printed Z-direction specimens, with differences generally falling between 1.40% and 7.10%. From the anisotropy index results, the 0.1-0.2-0% group exhibited relatively higher anisotropy, whereas the differences among the other mixtures were not significant.

#### 3.1.3. Flexural Strength

**Figure 12:**
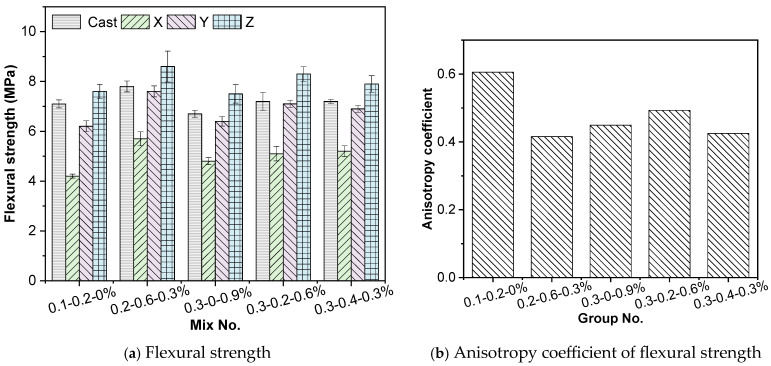
Flexural strength test results.

The flexural strength results of the specimens are presented in Figure 12. Pronounced anisotropy is observed in the 3D printed concrete under different loading directions. The Z-direction (layer deposition direction) exhibited the highest flexural strength, followed by the Y-direction, while the X-direction (parallel to the filament path) showed the lowest values. This trend can be attributed to the printing process: the compaction induced by the printing nozzle and the self-weight of subsequent layers increase the density and reduce the porosity in the Z-direction, resulting in higher bending resistance compared to the Y-direction.

Compared with cast specimens, the flexural strength in the Z-direction was 7.0%, 10.3%, 11.9%, 15.3%, and 10.1% higher, respectively. In contrast, the X-direction flexural strength at 28 days was significantly lower than that of cast specimens by 26.9%, 36.8%, 28.4%, 29.2%, and 27.8%, respectively. The anisotropy indices for flexural strength were all above 0.40, indicating that although the absolute strength differences among mixtures were not large, the degree of flexural anisotropy remained high for all groups.

### 3.2. Carbon Emissions of 3D Printed Low-Carbon Concrete

#### 3.2.1. Raw Material Production Stage

The carbon emission factors for the raw materials used in this study are listed in Table 4. Based on the GB/T 51366-2019 standard [38], the China Product Carbon Footprint Database (CPCD), and emission factors reported in the literature [39,40,41,42], the carbon emission factors for OPC, FA, SS, SF, RS, ITS, PP fibres, and SP were determined to be 0.95, 0.008, 0.0573, 0.014, 0.0051, 0.003, 1.85, and 0.75 kg CO_2_/kg, respectively. Table 4 summarizes the material consumption and corresponding carbon emissions for each 3D printed low-carbon concrete mixture.

As shown in Table 4, the carbon emissions of the mixtures range from 0.125 to 0.197 kgCO_2_. Higher cement and fibre contents resulted in greater emissions, whereas increasing the replacement ratios of steel slag and iron tailings sand led to lower emissions. This is mainly because steel slag is an industrial by-product of steelmaking and does not undergo high-temperature calcination or other energy-intensive processes required for cement production, resulting in a much lower emission factor. Therefore, replacing cement with steel slag significantly reduces the carbon emissions associated with raw materials. River sand mining involves excavation and transportation, both of which consume substantial amounts of energy and generate considerable carbon emissions. For example, extracting 1 m^3^ of river sand using heavy excavators requires approximately 10 L of diesel, producing about 26 kg of CO_2_. In contrast, iron tailings sand is a waste product generated from mineral processing. Replacing river sand with iron tailings sand eliminates the carbon emissions associated with sand extraction and transportation, thereby further reducing the carbon footprint of concrete production.

#### 3.2.2. Raw Material Transportation Stage

Table 5 summarizes the transport distances and emission factors of the raw materials. Carbon emissions vary with transportation mode; for example, heavy-duty trucks have an emission factor of 1.11 × 10^−4^ kgCO_2_/(kg·km), whereas medium-duty trucks have a higher factor of 2.35 × 10^−4^ kgCO_2_/(kg·km) [43]. The material quantities and corresponding transportation emissions for each concrete mixture are listed in Table 6.

#### 3.2.3. Concrete Mixing Stage

The average electricity consumption for producing one unit mass of concrete is 0.002 kWh, with an associated emission factor of 0.606 kgCO_2_/kWh [44]. Thus, the carbon emissions generated in the concrete mixing stage amount to 0.00121 kgCO_2_ per unit mass of concrete.

#### 3.2.4. Carbon Emission Results and Analysis

The production of 3D printed low-carbon concrete involves three major stages: raw material production, material transportation, and concrete mixing. The total carbon emissions for each mixture are presented in Table 7.

From a full-process perspective, emissions from raw material production account for 93.48–96.32% of the total carbon emissions associated with concrete production. In comparison, emissions from transportation and mixing collectively contribute less than 10%. This clearly indicates that raw material production is the dominant factor influencing the carbon footprint of 3D printed concrete. Accordingly, optimizing the material composition serves as the most effective approach for carbon reduction.

Replacing cement with steel slag and replacing river sand with tailings sand can substantially reduce overall emissions, aligning well with sustainable construction principles. Emissions from material transportation, although comparatively minor, can be further reduced by improving logistics planning, adopting low-carbon transportation modes, and sourcing materials locally. While the contributions of electricity consumption in mixing and fuel consumption during transport are relatively small, process optimization and efficient planning can still yield meaningful reductions. Together with mix-design optimization, these strategies form a comprehensive carbon-reduction framework across the entire production cycle.

### 3.3. Comprehensive Performance Evaluation of 3D Printed Low-Carbon Concrete Based on TOPSIS

Given that multiple performance indicators are involved in this study and that their impacts on the evaluation outcome vary significantly, an objective weighting method is required to quantify the contribution of each indicator within the comprehensive assessment framework. For reusable formwork applications, 3D printed low-carbon concrete must possess sufficient mechanical strength while exhibiting low environmental impact. Therefore, an integrated evaluation system considering printability, mechanical properties, and carbon performance was established.

To achieve a systematic assessment of different mixtures, four key indicators, compressive strength, flexural strength, splitting tensile strength, and carbon emissions per unit volume, were selected to construct a multi-dimensional performance evaluation model. The coefficient-of-variation TOPSIS (Technique for Order Preference by Similarity to Ideal Solution) method was applied to rank the overall performance of the five mixtures [45,46].

The coefficient-of-variation method is an objective statistical weighting approach. It assigns weights based on the dispersion of indicator data; indicators with greater variability receive higher weights because they contribute more to distinguishing among samples. This data-driven weighting strategy eliminates subjective bias and reflects the actual influence of each indicator on performance evaluation. Taking five alternatives and four evaluation criteria as an example in this study, the procedure is summarized as follows.

An initial decision matrix X = (*x_ij_*)_5×4_ was first established, where *x_ij_* denotes the value of the *i*-th alternative under the *j*-th criterion, and all criteria were unified to a benefit-type form. To eliminate dimensional effects, vector normalization was applied as $z_{ij}=x_{ij}/\sqrt{\sum_{i=1}^{5}x_{ij}^{2}}$, yielding the normalized matrix Z. The coefficient of variation was then employed to objectively determine criterion weights, with ${CV}_{j}=s_{j}/{\bar{z}}_{j}$ and $w_{j}={CV}_{j}/\sum_{j=1}^{4}{CV}_{j}$, where ${\bar{z}}_{j}$ and $s_{j}$ are the mean and standard deviation of the normalized values, respectively. The weighted normalized matrix was obtained by $v_{ij}=w_{j}z_{ij}$. Subsequently, the positive and negative ideal solutions were defined as $Y^{+}=\left({max}_{i}v_{ij}\right)$ and $Y^{-}=\left({min}_{i}v_{ij}\right)$. The Euclidean distances of each alternative from the ideal solutions were calculated using $D_{i}^{+}=\sqrt{\sum_{j=1}^{4}{\left(v_{ij}-Y_{j}^{+}\right)}^{2}}$ and $D_{i}^{-}=\sqrt{\sum_{j=1}^{4}{\left(v_{ij}-Y_{j}^{-}\right)}^{2}}$, and the closeness coefficient was finally determined as $C_{i}=D_{i}^{-}/\left(D_{i}^{+}+D_{i}^{-}\right)$. Alternatives with larger *C_i_* values were considered to exhibit superior overall performance.

In line with the design requirements, compressive strength, flexural strength, and splitting tensile strength were treated as benefit attributes, whereas carbon emissions, being a cost attribute, were converted to a benefit indicator by taking the reciprocal. The calculated weights are presented in Table 8.

As shown in Table 8, carbon emissions hold the greatest weight, followed by splitting tensile strength, while compressive and flexural strengths have the lowest weights. This ranking is reasonable: all mixtures exhibit adequate compressive and flexural capacities for formwork applications; thus, their discriminative significance is limited. Splitting tensile strength reflects the interlayer bond quality, which is essential to prevent sliding failure when subjected to lateral pressure during casting, and therefore carries a higher weight. Once mechanical requirements are met, the environmental impact becomes the dominant concern, explaining why carbon emissions receive the highest weight.

The positive and negative ideal solutions calculated by the TOPSIS model are: Positive ideal solution: Y^+^ = (0.104, 0.107, 0.047, 0.237), and Negative ideal solution: Y^−^ = (0.084, 0.088, 0.043, 0.155).

Based on the coefficient-of-variation-weighted TOPSIS evaluation, the five mixture proportions are ranked as 0.3-0.2-0.6% (*C_i_* = 0.8823) ≈ 0.3-0.4-0.3% (*C_i_* = 0.8803) > 0.3-0-0.9% > 0.2-0.6-0.3% > 0.1-0.2-0%, see Table 9. Although the mixture of 0.3-0.2-0.6% achieves the highest closeness coefficient, its comprehensive performance is very close to that of the 0.3-0.4-0.3%, indicating no substantial difference in overall evaluation. From the perspectives of resource utilization and engineering economics, the 0.3-0.4-0.3% mixture allows a higher substitution level of iron tailings sand while reducing fibre dosage, which is beneficial for large-scale utilization of solid waste and effective material cost control. Considering both the TOPSIS evaluation results and practical engineering factors, the mixture with a ratio of 0.3-0.4-0.3% is therefore recommended as the optimal mixture design under the investigated conditions.

## 4. Finite Element Modelling

To evaluate the feasibility of the proposed connection strategies, ABAQUS (version 2020) finite element analysis was employed to simulate the structural performance of the curved wall formwork under the lateral pressure exerted by fresh concrete. Strength control: The formwork material was the optimized 3D printed low-carbon concrete identified in this study. Since the formwork primarily bears lateral pressure in the Y-direction, the design compressive strength in this direction was taken as *f_cu_* = 45.3 MPa. Deflection control: In the absence of relevant design specifications for 3D printed concrete formwork, the deflection limit was determined by referencing steel formwork design criteria. According to these specifications, the maximum allowable deflection is 1/400, corresponding to 1.5 mm for the present formwork span. Therefore, the allowable deflection of the 3D printed concrete formwork was set to 1.5 mm.

### 4.1. Model Description

**Figure 13:**
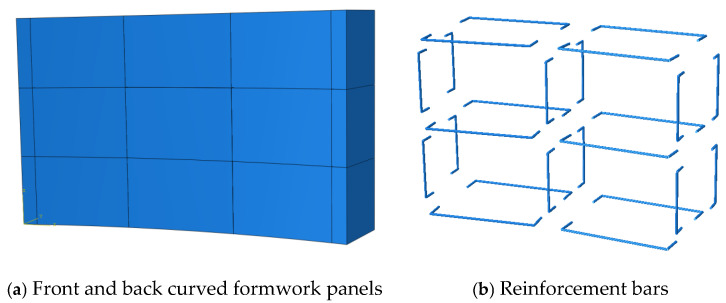
Three-Dimensional printed concrete formwork modelling components.

The finite element model was constructed using a part-based modelling approach. The reusable 3D printed concrete formwork developed in this study consists of two printed curved concrete panels (front and back) and internal reinforcement. Pre-printing trials confirmed that the printed formwork exhibits strong overall integrity and does not undergo interlayer sliding during concrete casting. Thus, to simplify the modelling process, the formwork was treated as a monolithic component, and the interlayer surface irregularities were neglected. The geometry of each component in the finite element model is shown in Figure 13.

In the Property module of ABAQUS, the material properties, section definitions, and material assignments were completed. Since the lateral pressure applied during casting keeps the formwork within the elastic range, an elastic constitutive model was adopted to improve computational efficiency. Based on preliminary tests, the elastic modulus of the printed concrete was set to 36,000 MPa, with axial compressive and tensile strengths of 45.3 MPa and 2.85 MPa, respectively, and a Poisson’s ratio of 0.2. The reinforcing steel was assigned an elastic modulus of 210 GPa and a yield strength of 400 MPa.

Using the Assembly module, the concrete formwork and reinforcing bars were assembled. The external concrete panel was modelled using C3D8R solid elements, while the reinforcement was represented by T3D2 truss elements. Mechanical interactions among components were defined in the Interaction module, ensuring correct load transfer during analysis. With respect to the steel–concrete interface modelling, an embedded region constraint was applied to embed the extended segments of the U-shaped reinforcing bars into the concrete matrix, assuming perfect bond. This assumption is considered reasonable, as the analysis focuses on global stiffness and deformation response under service-level lateral pressure, rather than local bond-slip behaviour.

**Figure 14:**
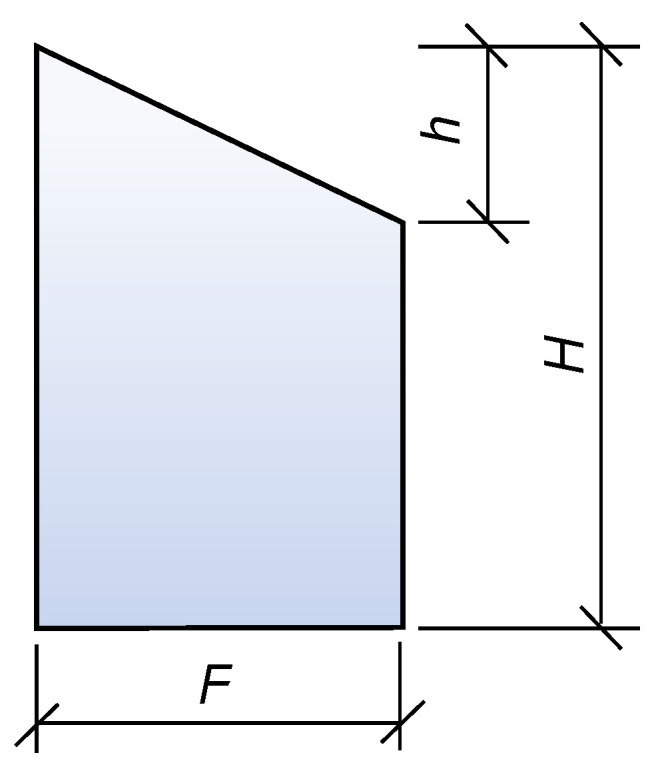
Schematic of concrete lateral pressure distribution.

Boundary conditions were applied by fully constraining the base of the formwork to prevent rigid body motion, while allowing deformation elsewhere. The self-weight of the formwork was introduced through gravity loading, and the lateral pressure of freshly cast concrete was applied as a linearly varying distributed load along the height of the formwork, consistent with relevant design codes and practical casting conditions. The lateral pressure and self-weight produced by freshly cast concrete were applied according to GB 50666-2011 [47] and GB 50204-2002 [48]. The pressure distribution is shown in Figure 14, where the designed formwork height is *H* = 0.9 m, the effective head height is *h* = 0.43 m, and the standard lateral pressure is 10.23 kN/m^2^. After defining the material properties and element types, the finite element model was discretized using a structured mesh. A preliminary mesh sensitivity check indicated that mesh sizes of 10 mm and 20 mm produce nearly identical stress and displacement results, whereas further coarsening beyond 20 mm leads to a noticeable reduction in accuracy. Accordingly, a mesh seed size of 20 mm was adopted as a compromise between numerical accuracy and computational efficiency. Since the modelling procedures of the three connection strategies are largely similar, with differences mainly in formwork segmentation and reinforcement arrangement, only Strategy 1 is described in detail.

### 4.2. Strength Verification

**Figure 15:**
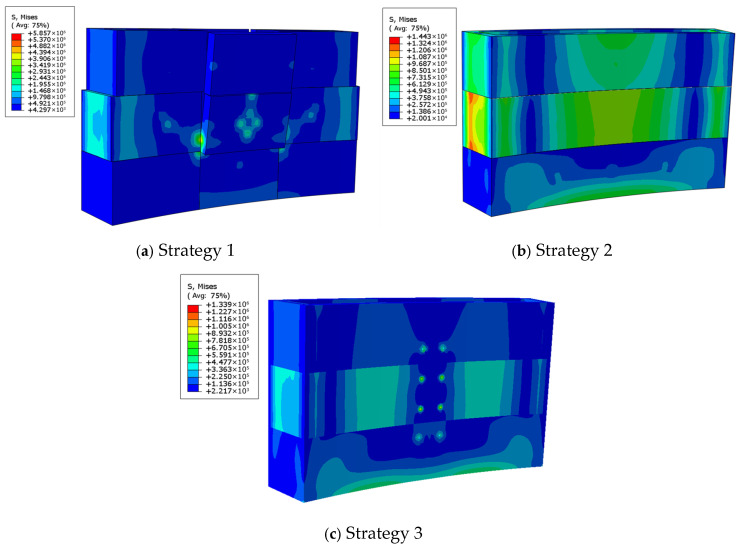
Stress analysis results of each strategy.

The stress distributions of the 3D printed reusable formwork under different connection strategies are shown in Figure 15. The finite element results indicate that the maximum stresses for Strategy 1, Strategy 2, and Strategy 3 are 5.86 MPa, 1.44 MPa, and 1.10 MPa, respectively. Higher stresses are observed near the mid-span sections where both ends require connection, and stress concentrations appear around corners and joint regions. Given the allowable concrete stress of 45.3 MPa, all three strategies satisfy the strength requirements. Among them, Strategy 1 exhibits the highest stress (5.86 MPa), whereas Strategy 3 shows the lowest (1.10 MPa).

### 4.3. Stiffness Verification

**Figure 16:**
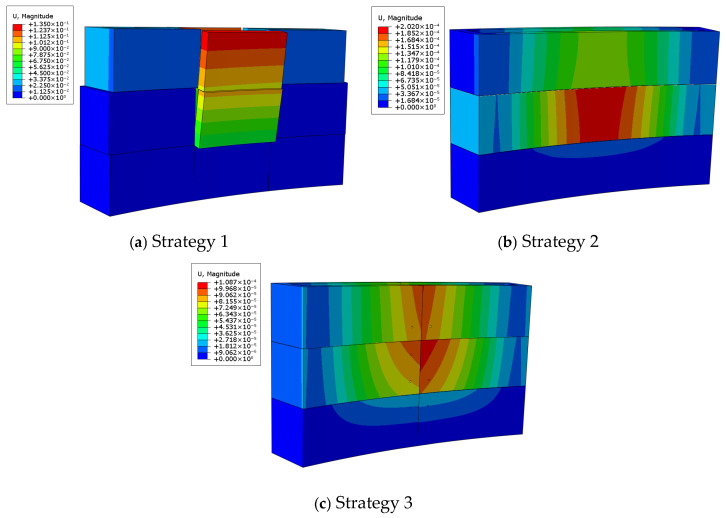
Displacement analysis results of each strategy.

The displacement contours for each connection strategy are presented in Figure 16. The maximum deflections obtained are 135 mm, 0.202 mm, and 0.027 mm for Strategy 1, Strategy 2, and Strategy 3, respectively. The allowable deflection for all strategies is 0.25 mm. The results show that Strategy 1 exhibits excessive deformation (135 mm) due to insufficient connection stiffness, which may cause leakage of fresh concrete during casting and thus fails to meet serviceability requirements. In contrast, both Strategy 2 and Strategy 3 satisfy the deflection limit, with Strategy 3 showing the best stiffness performance, reflected by its minimal deformation of only 0.027 mm.

### 4.4. Simulation Discussion

The preceding analysis indicates that Strategy 1, due to its structural configuration and material arrangement, fails to meet the deflection limits required by relevant design codes. Under the lateral pressure of fresh concrete during casting, this connection system is prone to excessive deformation, which may lead to dimensional deviations, or even severe failures such as formwork bulging or bursting, making it unsuitable for practical engineering applications.

**Figure 17:**
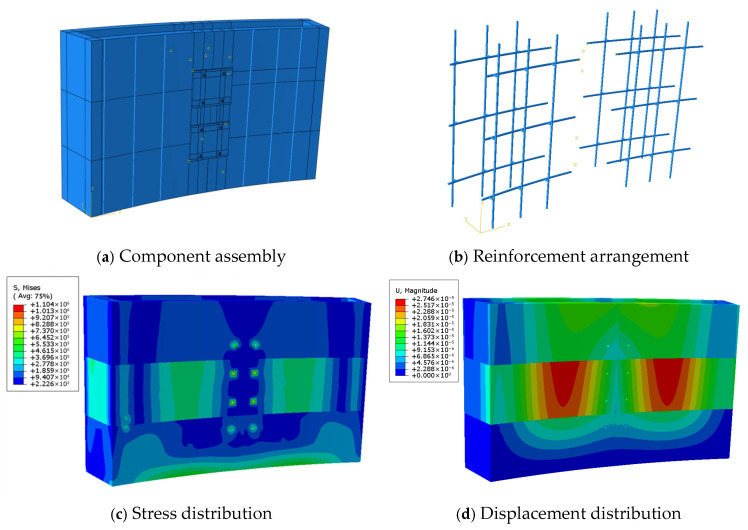
Finite element analysis results of the combined strategy.

In contrast, Strategies 2 and 3 exhibit better engineering adaptability. Depending on project requirements, structural geometry, and construction conditions, either strategy can be applied independently or combined. Figure 17 illustrates the performance of the combined strategy. Finite element results show that the maximum stress of the combined configuration is 1.10 MPa, representing a reduction of 23.61% compared with Strategy 2 and 16.67% compared with Strategy 3. The maximum displacement is 0.027 mm, which is reduced by 86.38% and 74.63% compared with Strategies 2 and 3, respectively. These results demonstrate that the combined strategy substantially enhances the overall stability of the formwork system and improves its resistance to lateral deformation and sliding, thereby ensuring construction quality and structural safety during concrete casting.

## 5. Conclusions

This study conducted a comprehensive evaluation of 3D printed low-carbon concrete, including its mechanical properties and production-stage carbon emissions. The coefficient-of-variation TOPSIS method was employed to identify the optimal mix design. Furthermore, three connection strategies for reusable decorative formwork were developed and analyzed through finite element simulations. The major conclusions are as follows:

(1) The mixtures showed clear mechanical anisotropy: compressive strength was highest in the X-direction, splitting and flexural strength peaked in the Z-direction, while the lowest values appeared in the X-direction. Higher steel slag and iron tailings sand replacement ratios led to notable strength improvements.
(2) Carbon emissions are dominated by raw material production, while transportation and mixing contribute minimally. Replacing cement with steel slag and natural sand with tailings sand is effective for emission reduction.
(3) Both the 0.3-0.2-0.6% and 0.3-0.4-0.3% mixtures demonstrate excellent overall performance through the coefficient-of-variation TOPSIS method. The 0.3-0.4-0.3% mixture is recommended for practical application in 3D printed low-carbon concrete, as it achieves a favourable balance between mechanical strength and low-carbon objectives by increasing iron tailings sand utilization while reducing fibre content.
(4) All three connection strategies meet strength requirements, but Strategy 1 fails the deflection limit. Strategies 2 and 3 satisfy stiffness criteria, with Strategy 3 showing the smallest deformation. A combined use of Strategies 2 and 3 further enhances system stability, offering practical guidance for engineering application of reusable 3D-printed formwork.

While this study focuses on mechanical performance, carbon efficiency, and structural applicability, future work will systematically investigate the durability performance of 3D-printed concrete incorporating steel slag and iron tailings, such as sulphate resistance, carbonation, freeze–thaw behaviour, and moisture transport across printed layers.

## Figures and Tables

**Table 1:** Nominal physical properties of PP fibre.

Fibre	Density (g/cm^3^)	Length (mm)	Average Diameter (µm)	Tensile Strength (MPa)	Elastic Modulus (GPa)	Rupture Elongation (%)
PP	0.91	6	18	500	5	15

**Table 2:** Mix proportions of 3D printable concrete (wt.%).

Mix No.	OPC	SS	FA	SF	RS	ITS	PP	W	SP
0.1-0.2-0%	0.5	0.1	0.3	0.1	1.2	0.3	0%	0.3	0.4%
0.2-0.6-0.3%	0.4	0.2	0.3	0.1	0.6	0.9	0.3%	0.3	0.4%
0.3-0-0.9%	0.3	0.3	0.3	0.1	1.5	0	0.9%	0.3	0.4%
0.3-0.2-0.6%	0.3	0.3	0.3	0.1	1.2	0.3	0.6%	0.3	0.4%
0.3-0.4-0.3%	0.3	0.3	0.3	0.1	0.9	0.6	0.3%	0.3	0.4%

Note: OPC: Ordinary Portland cement; RS: River sand; ITS: Iron tailings sand; W: Water; SP: Superplasticizer; PP fibre: Polypropylene fibre. All numbers are mass ratios of the binder mass, except the PP content (volume fraction).

**Table 3:** Printability test results.

Mix No.		0.1-0.2-0%	0.2-0.6-0.3%	0.3-0-0.9%	0.3-0.2-0.6%	0.3-0.4-0.3%
Extrudability test	Appearance	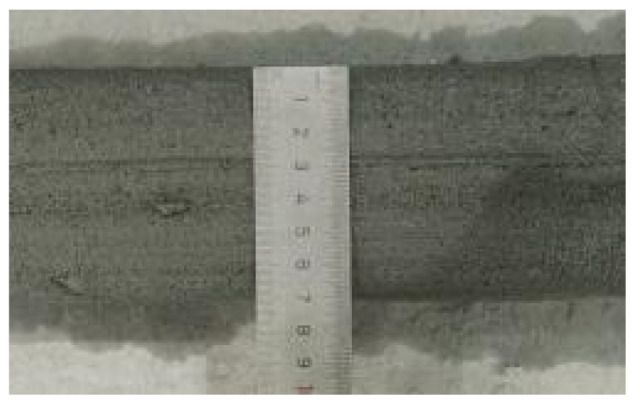	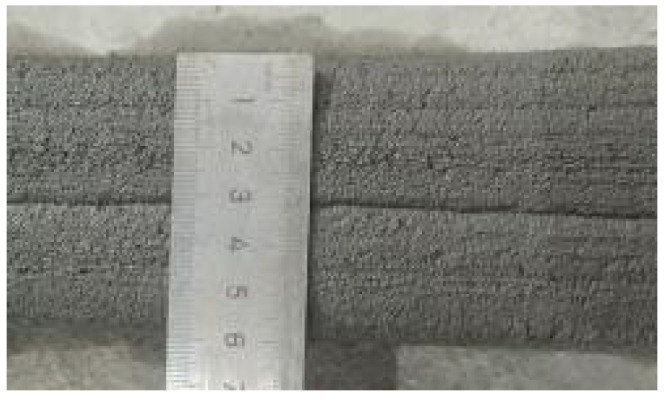	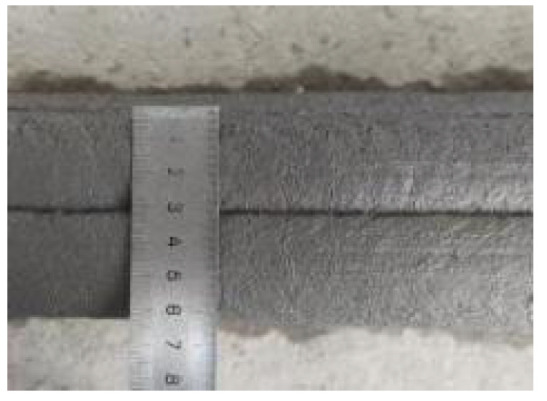	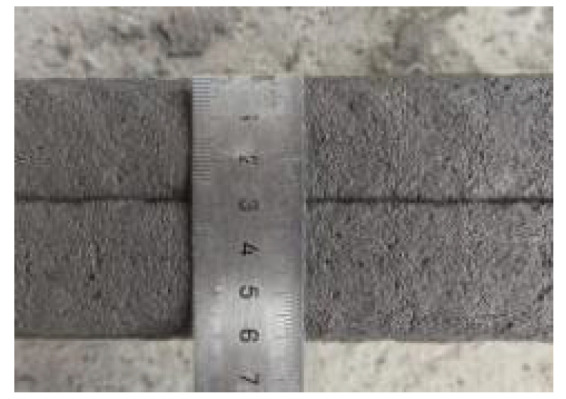	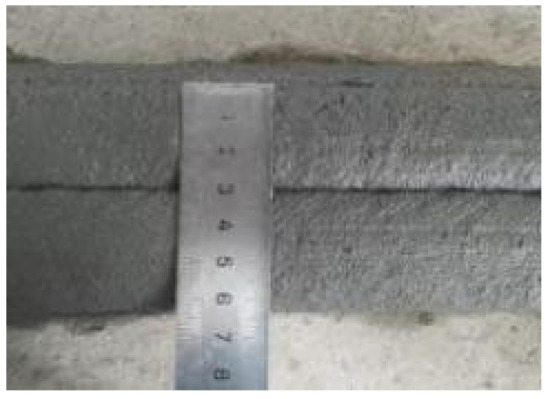
	Width	66 mm	59 mm	62 mm	61 mm	63 mm
Buildability testing	Appearance	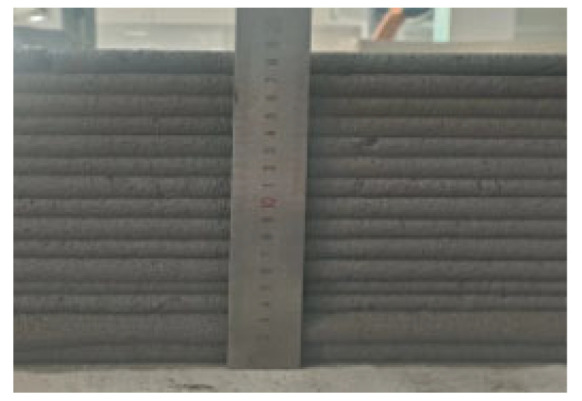	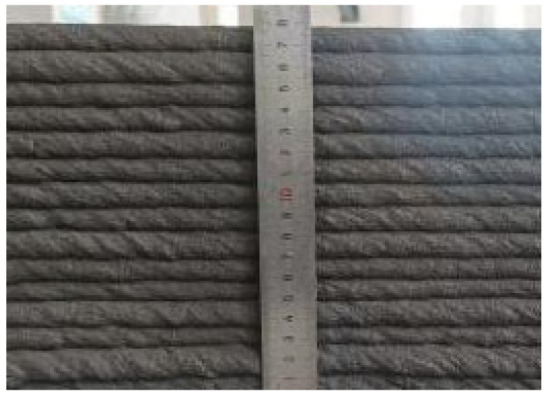	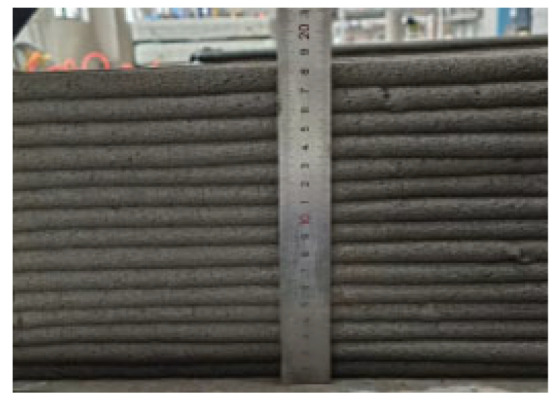	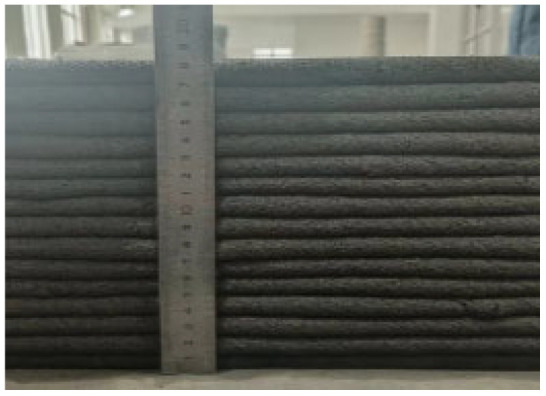	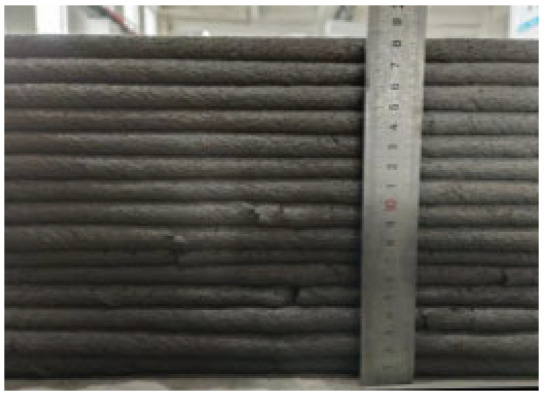
	Height	184.2 mm	178.8 mm	183.1 mm	182.3 mm	183.0 mm
	Average deformation rate	2.3%	0.6%	1.7%	1.3%	1.7%

**Table 4:** Unit material consumption and carbon emissions of raw materials in the production stage.

Mix No.	OPC (kg)	FA (kg)	SS (kg)	SF (kg)	RS (kg)	ITS (kg)	PP (kg)	SP (kg)	Carbon Emissions (kgCO_2_)
0.1-0.2-0%	0.2	0.12	0.04	0.04	0.48	0.12	0	0.0016	0.197
0.2-0.6-0.3%	0.16	0.12	0.08	0.04	0.24	0.36	0.0002	0.0016	0.162
0.3-0-0.9%	0.12	0.12	0.12	0.04	0.6	0	0.0005	0.0016	0.130
0.3-0.2-0.6%	0.12	0.12	0.12	0.04	0.48	0.12	0.0003	0.0016	0.127
0.3-0.4-0.3%	0.12	0.12	0.12	0.04	0.36	0.24	0.0002	0.0016	0.125

**Table 5:** Transportation distances of raw materials and corresponding carbon emission factors.

Raw Material	OPC	FA	SS	SF	RS	ITS	PP	SP
Transport distance (km)	30	30	20	30	50	60	20	20
Carbon emission factor (10^−4^ kgCO_2_/kg × km)	1.1	2.35	2.35	2.35	1.11	2.35	2.35	2.35

**Table 6:** Unit carbon emissions during the raw material transportation stage.

Mix No.	OPC (kg)	FA (kg)	SS (kg)	SF (kg)	RS (kg)	ITS (kg)	PP (kg)	SP (kg)	Carbon Emissions (kgCO_2_)
0.1-0.2-0%	0.2	0.12	0.04	0.04	0.48	0.12	0	0.0016	0.0063
0.2-0.6-0.3%	0.16	0.12	0.08	0.04	0.24	0.36	0.0002	0.0016	0.0084
0.3-0-0.9%	0.12	0.12	0.12	0.04	0.6	0	0.0005	0.0016	0.0054
0.3-0.2-0.6%	0.12	0.12	0.12	0.04	0.48	0.12	0.0003	0.0016	0.0064
0.3-0.4-0.3%	0.12	0.12	0.12	0.04	0.36	0.24	0.0002	0.0016	0.0075

**Table 7:** Summary of unit carbon emissions during the concrete production stage (unit: kgCO_2_).

Mix No.	Raw Material Production (*C*_11_)	Raw Material Transportation (*C*_12_)	Concrete MIXING Process (*C*_13_)	Concrete Production (*C*_1_)
0.1-0.2-0%	0.197	0.0063	0.00121	0.20451
0.2-0.6-0.3%	0.162	0.0084	0.00121	0.17161
0.3-0-0.9%	0.130	0.0054	0.00121	0.13661
0.3-0.2-0.6%	0.127	0.0064	0.00121	0.13461
0.3-0.4-0.3%	0.125	0.0075	0.00121	0.13371

**Table 8:** Weights of evaluation indicators for 3D-printed low-carbon concrete.

Evaluation Indicator	Mean	Standard Deviation	Coefficient of Variation *CV_j_*	Weight *w_j_*
Compressive strength (MPa)	43.28	3.13	0.072	0.211
Flexural strength (MPa)	6.84	0.50	0.074	0.217
Splitting tensile strength (MPa)	3.07	0.10	0.034	0.100
Carbon emissions (kgCO_2_/^−1^)	6.59	1.05	0.161	0.472

**Table 9:** TOPSIS evaluation results.

Mix No.	0.1-0.2-0%	0.2-0.6-0.3%	0.3-0-0.9%	0.3-0.2-0.6%	0.3-0.4-0.3%
*C_i_*	0.0023	0.4407	0.7745	0.8823	0.8803
Ranking	5	4	3	1	2

## Data Availability

The original contributions presented in this study are included in the article. Further inquiries can be directed to the corresponding authors.
